# Exploiting Ligand-Protein Conjugates to Monitor Ligand-Receptor Interactions

**DOI:** 10.1371/journal.pone.0037598

**Published:** 2012-05-31

**Authors:** Hirohito Haruki, Monica Rengifo Gonzalez, Kai Johnsson

**Affiliations:** École Polytechnique Fédérale de Lausanne (EPFL), Institute of Chemical Sciences and Engineering, Institute of Bioengineering, National Centre of Competence in Research (NCCR) in Chemical Biology, Lausanne, Switzerland; Institute of Enzymology of the Hungarian Academy of Science, Hungary

## Abstract

We introduce three assays for analyzing ligand-receptor interactions based on the specific conjugation of ligands to SNAP-tag fusion proteins. Conjugation of ligands to different SNAP-tag fusions permits the validation of suspected interactions in cell extracts and fixed cells as well as the establishment of high-throughput assays. The different assays allow the analysis of strong and weak interactions. Conversion of ligands into SNAP-tag substrates thus provides access to a powerful toolbox for the analysis of their interactions with proteins.

## Introduction

Methods for the detection of ligand-receptor interactions are a crucial part of drug discovery and chemical biology in general [1]–[5]. For the identification of the protein targets of a given ligand (i.e. a drug or bioactive small molecule), affinity chromatography is most commonly used [6]. If the purpose is the identification of binders to a given protein, high-throughput-compatible approaches such as radioisotope-, fluorescence- or luminescence-based detection methods are preferred [2], [3]. For a detailed biophysical characterization of a known ligand-receptor interaction, approaches such as isothermal titration calorimetry, surface plasmon resonance, NMR or X-ray crystallography are chosen [4]. However, additional factors such as availability, purity, solubility, and stability of the protein of interest influence the assay choice. Overall, the development of suitable methods for the detection of ligand-receptor interactions can still be a formidable challenge and the availability of tools to rapidly establish a variety of complementary assay systems would help to overcome this challenge.

**Figure 1:**
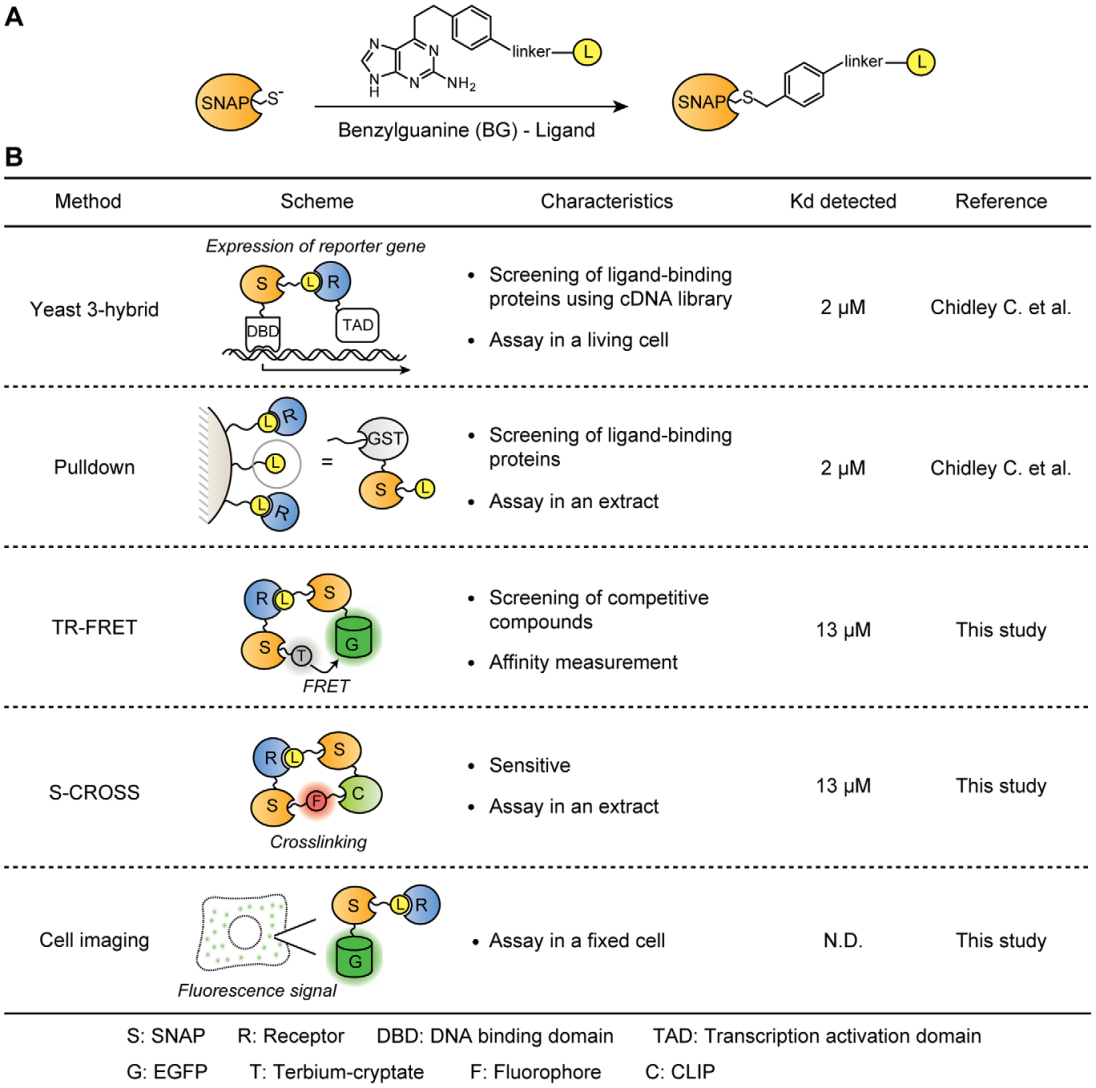
SNAP-based toolbox for detection and analysis of ligand-receptor interactions. (A) Covalent labeling of SNAP-tag with a ligand using a BG derivative. (B) Schematic representation of the different SNAP-based methods. The highest Kd values detected in this study using the pairs of MTX-eDHFR (WT and mutants) are presented for each method.

We recently introduced a SNAP-tag-based yeast three-hybrid system for the identification of protein targets of drugs and bioactive small molecules [7]. The approach is based on the derivatization of a ligand with benzylguanine (BG); BG derivatives can be specifically coupled to SNAP-tag fusion proteins in living cells (Figure 1A) [8]. Coupling of the ligand to an appropriate SNAP-tag fusion protein in yeast permits the screening of cDNA libraries for its protein targets (Figure 1B) [7]. Furthermore, by coupling ligands to SNAP-tag fusions that can be specifically immobilized on beads, we also established a SNAP-based pulldown assay using mammalian cell extracts (Figure 1B) [7]. Here we build on these results and demonstrate how the coupling of BG derivatives of ligands to different SNAP-tag fusion proteins can be exploited to rapidly establish different assays based on either time-resolved fluorescence resonance energy transfer (TR-FRET) [9], selective crosslinking (S-CROSS) [10], or fluorescence microscopy [11]. Together, these methods form a powerful toolbox for the identification and analysis of ligand-receptor interactions based on a single ligand derivative.

## Results

### SNAP-based TR-FRET Assay

**Figure 2:**
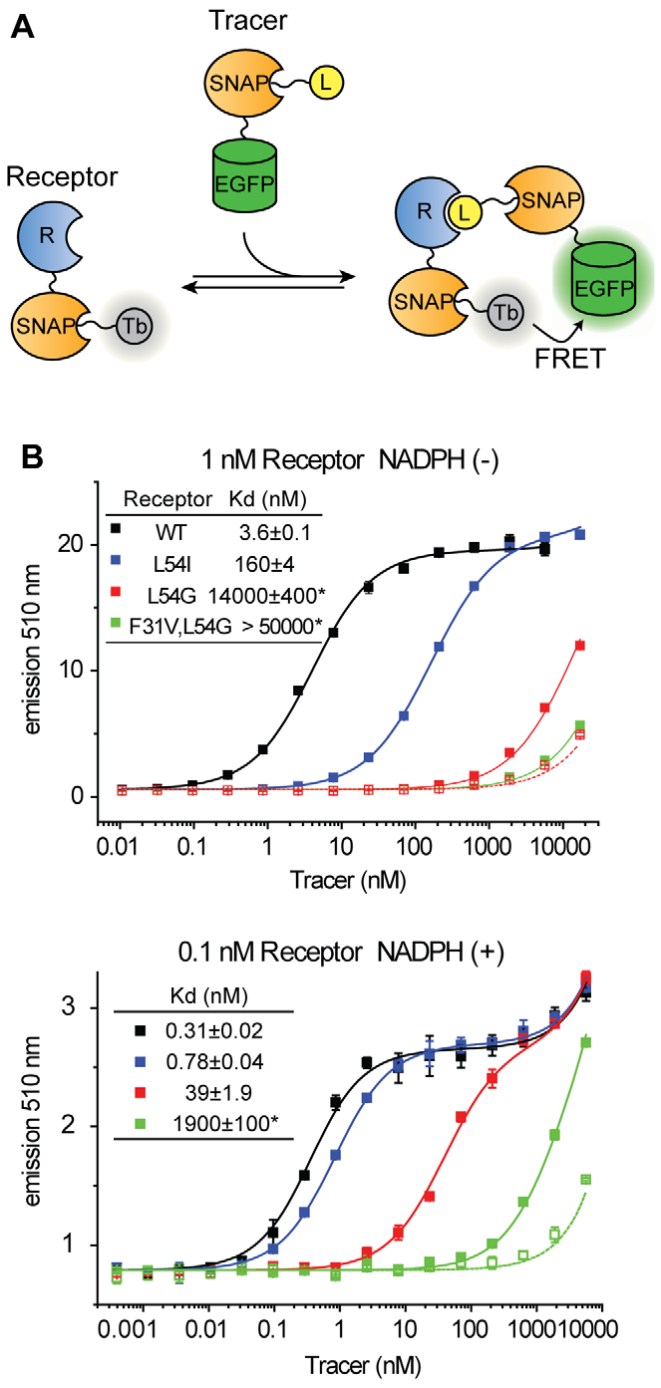
SNAP-based TR-FRET titration assay. (A) Scheme of the titration binding assays. The titration assay measures the affinity of the tracer for the receptor. (B) Titration assay using MTX-SNAP-EGFP (filled rectangle) or SNAP-EGFP (empty rectangle) as tracer and SNAP-eDHFR as receptor in the absence and presence of 100 µM NADPH. SNAP-eDHFR is 50% labeled with BG-Terbium cryptate (Tb). Representative data using receptor concentration of 1 nM (in the absence of NADPH) and 0.1 nM (in the presence of NADPH) are shown. The specific receptor concentration was chosen so that it was below the Kd of the analyzed interaction. Kd values and the standard error of the mean are shown in the graph. * indicates that the Kd values were calculated with Fmax of the higher affinity samples (see Materials and Methods).

**Figure 3:**
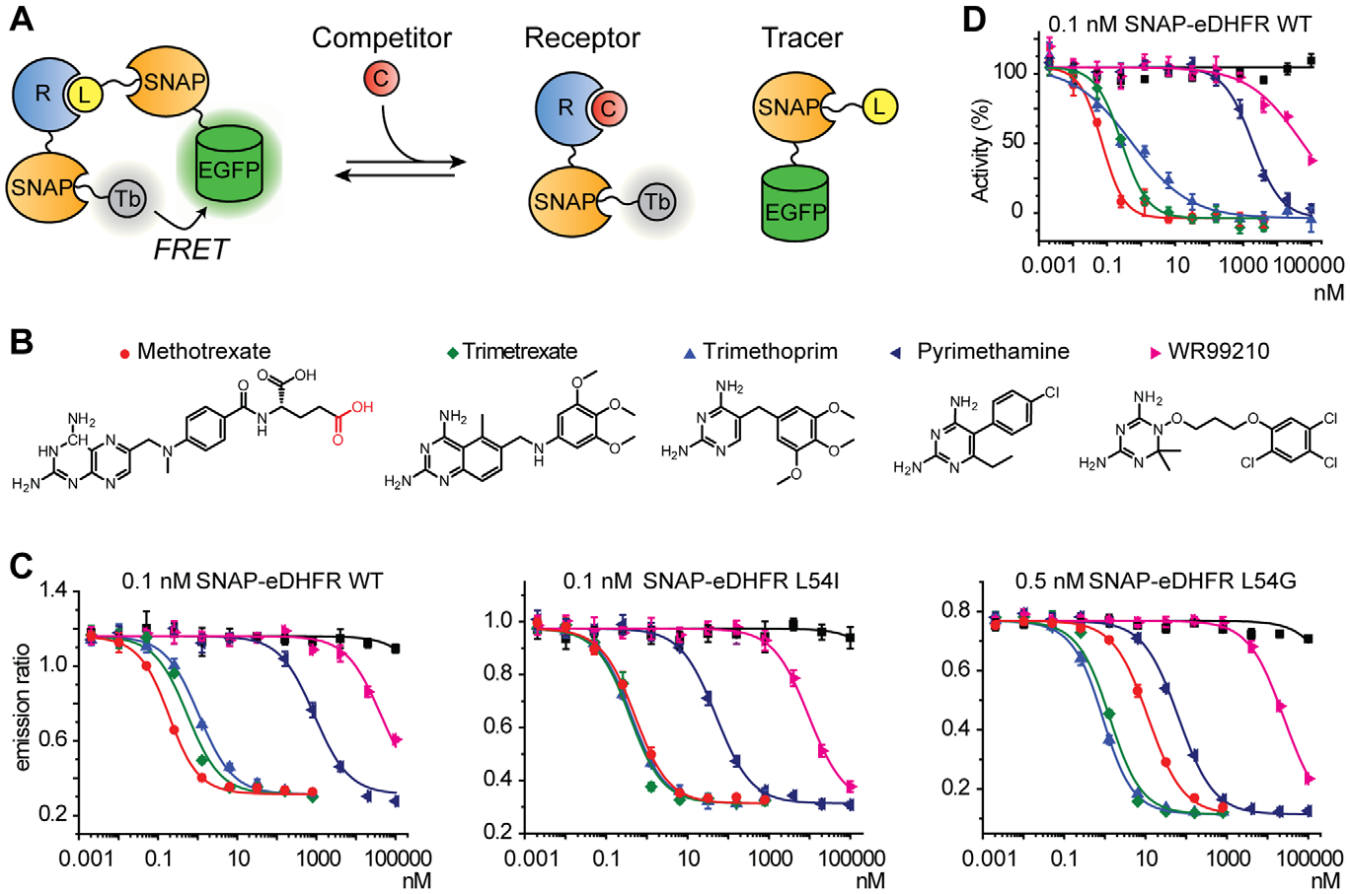
SNAP-based TR-FRET competition assay. (A) Scheme of the competition binding assay. The competition assay measures the affinity of the ligand for the receptor. (B) Chemical structures of DHFR inhibitors. MTX was linked to BG via the carboxyl group highlighted in red. (C) SNAP-based TR-FRET competition assays using indicated concentrations of SNAP-eDHFR WT, L54I and L54G. Concentrations of MTX-SNAP-EGFP are 1 nM, 2 nM, and 30 nM for SNAP-eDHFR WT, L54I and L54G, respectively. Rectangle filled with black indicates dilution series of DMSO. Maximum DMSO concentration is 0.5% at 100 µM compounds. (D) DHFR enzymatic activity inhibition assay using SNAP-eDHFR WT.

To develop a method for the quantification of ligand-receptor interactions that is also suitable for high-throughput applications, we took advantage of both SNAP-tag and TR-FRET technology using lanthanides (Figure 1B). By combining these two technologies, one can measure the affinity of both derivatized ligand and free ligand for the receptor by simple titration (Figure 2) and competition experiments (Figure 3), respectively. This assay is highly sensitive, and is a simple “mix and measure” protocol without washing step, which makes it easily applicable for a high-throughput format.

In the SNAP-tag-based TR-FRET assay, as shown in Figure 2A, a ligand was conjugated via SNAP-tag to EGFP acting as FRET acceptor (tracer). Terbium-cryptate (Tb) connected to the receptor protein via SNAP-tag formed the FRET donor [12], [13]. The excitation of Tb at a wavelength of 340 nm induces the emission of fluorescence of long life-time (micro- to milliseconds) at a maximum of 490 nm. Only when Tb and EGFP are in spatial proximity (below 10 nm), EGFP emits fluorescence at a maximum wavelength of 510 nm due to FRET.

To evaluate the performance of the method, we analyzed the binding of methotrexate (MTX) to *Escherichia coli* dihydrofolate reductase (eDHFR). The binding between MTX and eDHFR is well characterized and a series of eDHFR mutants are available, which bind to MTX with wide range of affinity [14], [15]. We prepared SNAP-tagged constructs of eDHFR wild-type (WT) and three eDHFR mutants (eDHFR L54I, eDHFR L54G and eDHFR F31V, L54G) for which the reported Kd values to free MTX range from low pM to low µM [16], [17]. The dissociation constant (Kd) of MTX-SNAP-EGFP (tracer) for SNAP-eDHFR (WT and mutants) was measured with a tracer titration binding assay (Figure 2A), using FRET signal readout and curve fitting based on a single site binding model [18]. To facilitate assay preparation, an excess of SNAP-eDFHR was incubated with BG-Tb and then directly used without further purification. Background signal was measured with SNAP-EGFP blocked with BG. The assay was performed in the absence or presence of saturating concentration of NADPH and at different concentrations of the receptor. Table 1 shows measured Kd values of SNAP-bound MTX. Representative binding data using 1 nM receptor (without NADPH) and 0.1 nM receptor (with 100 µM NADPH) are shown in Figure 2B. Kd values from 0.4 nM to 13 µM could be determined with this assay; the Kd between SNAP-bound MTX and eDHFR F31V, L54G in the absence of NADPH was too high to measure. The data clearly show that NADPH stabilizes the complex formation between MTX tracer and eDHFR WT (8-fold), as well as eDHFR L54I (180-fold), L54G (280-fold), or F31V, L54G (not calculated). It should be noted that background signal increased at tracer concentrations above 1 µM, especially at lower receptor concentration (Figure 2B). This background signal might be due to a nonspecific interaction either between Tb and the fluorophore, or between the tracer and the receptor.

**Table 1:** Dissociation constants of MTX-SNAP-EGFP for SNAP-eDHFR measured by SNAP-based TR-FRET titration assay.

NADPH	WT	L54I	L54G	F31V, L54G
−	3.3±0.6 nM	140±40 nM	13±1 µM*	>50 µM*
+	0.41±0.07 nM	0.76±0.4 nM	46±7 nM	1.9±0.1 µM*

Values are the mean±standard deviation (SD) from 2–6 independent experiments. Receptor concentrations used in each experiment in the absence of NADPH were 0.02, 0.3, 1 and 3 nM for WT, 0.3, 1 and 3 nM for L54I, 1 and 3 nM for L54G and F31V, L54G. Receptor concentrations used in the presence of NADPH were 0.02, 0.03, 0.1 and 0.3 nM for WT, 0.1, 0.3 and 0.5 nM for L54I, 0.1, 0.5 and 30 nM for L54G and F31V, L54G. * indicates that Kd values were calculated with Fmax of the higher affinity samples (see materials and methods).

### SNAP-based TR-FRET Competition Assay

The competition binding assay is based on the displacement of the tracer from the tracer-receptor complex by an unmodified compound, resulting in a decrease of the FRET signal (Figure 3A). Applications of the competition TR-FRET assay include measurement of Kd (or IC50) values of unlabeled ligands and high-throughput screening. To evaluate the SNAP-based TR-FRET assay in a competition binding mode, we analyzed the binding of five known DHFR inhibitors to eDHFR (Figure 3B): the anti-cancer drugs methotrexate and trimetrexate targeting human DHFR, the anti-malaria drugs WR99210 and pyrimethamine targeting plasmodium DHFR, and the anti-bacterial trimethoprim. Competition SNAP-based TR-FRET assay using MTX-SNAP-EGFP as tracer and SNAP-eDHFR as receptor were performed in the presence of saturating concentration of NADPH. Kd values were calculated using a single site competitive binding model of two different ligands to a receptor protein [19]. These measurements showed that trimetrexate, pyrimethamine and WR99210 are also inhibitors of eDHFR and the determined Kd values of the five inhibitors for SNAP-eDHFR WT ranged from Kd = 40 pM for methotrexate to Kd = 12 µM of WR99210 (Figure 3C; Table 2). Measurement of the Kd values of these DHFR inhibitors for SNAP-eDHFR mutants L54I and L54G revealed that Leu54 is important for methotrexate binding but not for the binding of other DHFR inhibitors (Table 2 and Figure 3C). Furthermore, the Kd values of MTX-BG were measured to examine the influence of the derivatization of MTX on its affinity for eDHFR (Figure S1). Notably, the derivatization of MTX with BG decreased its Kd values for every eDHFR mutant about 10-fold. The Kd values of MTX-tracer and MTX-BG for the SNAP-eDHFR mutants were similar even though the molecular weight of the tracer (SNAP-EGFP) is 52 kDa. We also determined the Z-factor of the assay, a parameter typically used to evaluate the suitability of an assay for high-throughput applications. Assays with Z-factors between 0.5–1 are considered to be very well suited for screening assays [20]. Under the conditions of Figure 3C, the Z-factor was 0.75 (0.1 nM eDHFR WT and 1 nM tracer), 0.71 (0.1 nM eDHFR L54I and 2 nM tracer), and 0.83 (0.5 nM L54G and 30 nM tracer), indicating that this assay is very well suited for high-throughput applications (Figure S2). Finally, we compared the results of the SNAP-based TR-FRET competition assay with those obtained via a classical DHFR activity assay (Figure 3D and Table 2). Measurement of IC50 values by an enzymatic assay using 0.1 nM SNAP-eDHFR WT confirmed the data of the SNAP-based TR-FRET competition assay.

**Table 2:** Dissociation constants of DHFR inhibitors measured by SNAP-based TR-FRET competition assay.

	Enzymatic assay IC50 (nM)WT	Kd (nM)WT	Kd (nM)L54I	Kd (nM)L54G
MTX	0.071±0.007	0.040±0.004	0.13±0.01	6.9±0.4
Trimetrexate	0.27±0.03	0.15±0.01	0.10±0.01	0.64±0.04
Trimethoprim	0.53±0.09	0.30±0.02	0.10±0.01	0.38±0.03
Pyrimethamine	1900±220	260±19	14±1	39±2
WR99210	54000±16000	12000±950	2600±210	15000±790

IC50 and Kd values are the mean±standard error (N = 3 and 4, respectively).

**Figure 4:**
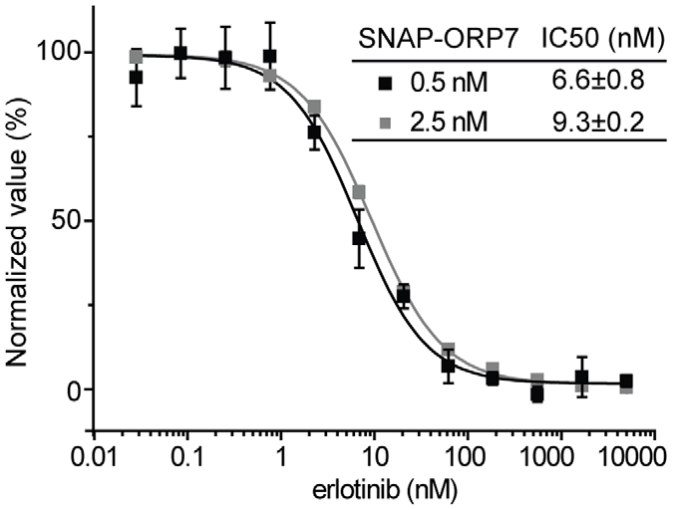
SNAP-based TR-FRET competition assay for ORP7-erlotinib interaction. The assay was performed at indicated concentrations of SNAP-ORP7 (receptor) and erlotinib-SNAP-EGFP (tracer) with erlotinib as a competitor. IC50 values and the standard error of the mean are shown.

Our next goal was the comparison of the performance of the SNAP-based TR-FRET assay with a more traditional fluorescence polarization (FP) assay. FP assays can be performed with unmodified receptor protein but necessitate the protein in higher concentrations. We used the previously identified interaction of the kinase inhibitor erlotinib with oxysterol-binding protein-related protein 7 (ORP7) to compare the two approaches [7]. We earlier measured its affinity by a FP assay using erlotinib derivatized with tetramethylrhodamine (TMR) and SNAP-ORP7. Titration and competition binding assays using 10 nM erlotinib-TMR revealed a Kd value of 230±3 nM, assuming single site binding, and an IC50 of 101±16 nM using erlotinib as a competitor, respectively [7]. Since the range of resolvable IC50 in a FP assay is limited by the affinity of the tracer due to ligand depletion [21], it is likely that the true IC50 of erlotinib for ORP7 is lower. The competition binding assay in SNAP-based TR-FRET using the same protein concentration (250 nM erlotinib-SNAP-EGFP for TR-FRET and 250 nM SNAP-ORP7 for FP assay) revealed that the IC50 value of erlotinib actually was 10 nM, one order of magnitude lower than the value measured by the FP assay (Figure 4). This demonstrates the superiority of TR-FRET assays over traditional FP assays for those receptor proteins that can be expressed as a (SNAP-tag) fusion protein. The measured IC50 furthermore confirms ORP7 as a non-kinase off-target of erlotinib.

### Sensitivity of SNAP-based Pulldown Assay

**Figure 5:**
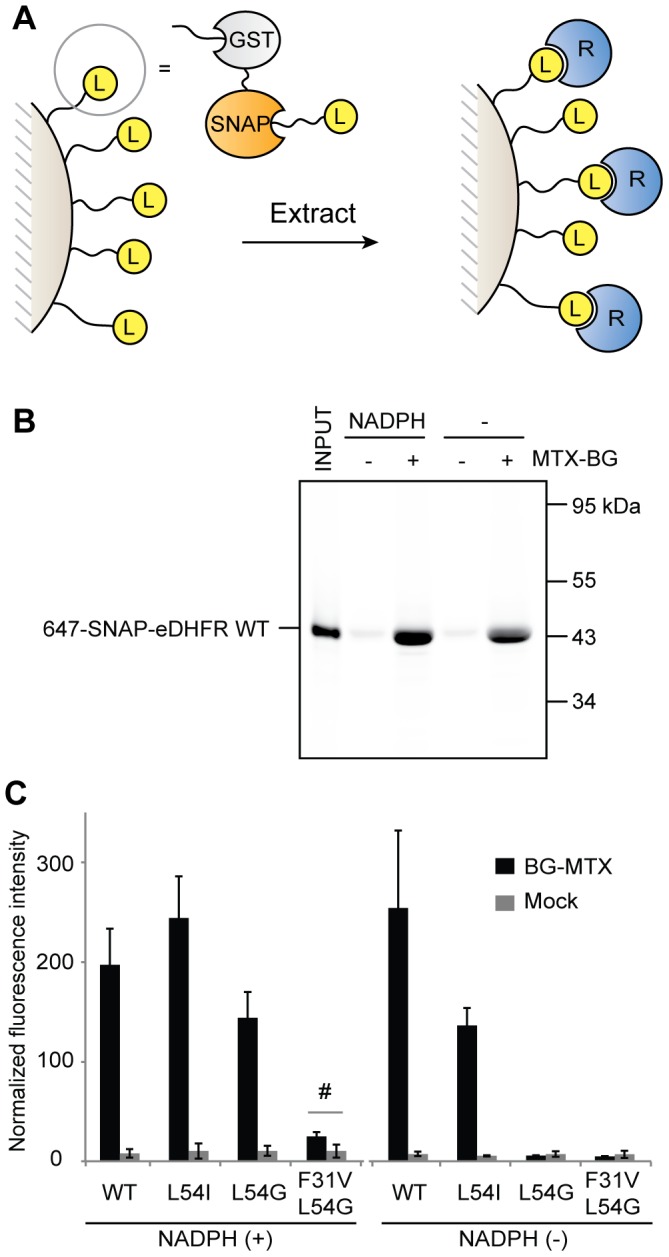
SNAP-based pulldown assay for detection of ligand-receptor interactions. (A) Scheme of the pulldown assay. The BG-ligand is immobilized on glutathione sepharose beads via GST-SNAP. Bound receptor proteins are concentrated on the beads after incubation with the extract and washing. (B) SNAP-eDHFR WT labeled with BG-647 (1 µM) was subjected to pulldown assay in the absence or presence of 100 µM NADPH using MTX immobilized on beads (MTX-BG +) or mock beads (MTX-BG -). Bound proteins were eluted with glutathione, submitted to SDS-PAGE and detected by in-gel fluorescence scanning. (C) Fluorescence signal of bound proteins normalized with the input signal in each gel (Mean±SD, n = 3). # represents P = 0.03 in paired t-test.

In the SNAP-based pulldown assay (Figures 1B, 5A), the BG-derivatized ligand is immobilized on glutathione-sepharose beads by means of SNAP-tag fused with glutathione S-transferase (GST) [7]. A pulldown is then performed using cell extract or purified protein. Bound proteins are eluted with glutathione, and subjected to SDS-PAGE analysis. The protein of interest can be then detected by Western blotting or other methods.

We examined the sensitivity of the SNAP-based pulldown assay with the SNAP-eDHFR mutants after preparation of MTX-immobilized beads using BG-MTX. For the quantitative analysis of bound proteins by in-gel fluorescence scanning after SDS-PAGE [10], SNAP-eDHFR was labeled with BG-647. The 647-SNAP-eDHFR (1 µM) was subjected to pulldown assay in the absence and presence of 100 µM NADPH. Figure 5B is a representative result of the pulldown assay using 647-SNAP-eDHFR WT, which bound specifically MTX immobilized on beads, irrespective of the presence or absence of NADPH. The fluorescence signal of bound 647-SNAP-eDHFR WT and mutants was quantified (Figure 5C), and the data showed that this assay is able to detect the interaction of MTX with the eDHFR F31V, L54G in the presence of NADPH, but not the interaction of MTX with eDHFR L54G in the absence of NADPH. Consequently, the upper detection limit of SNAP-based pulldown assay for this ligand-receptor pair is in the range of Kd values around 2 µM.

### S-CROSS Assay

**Figure 6:**
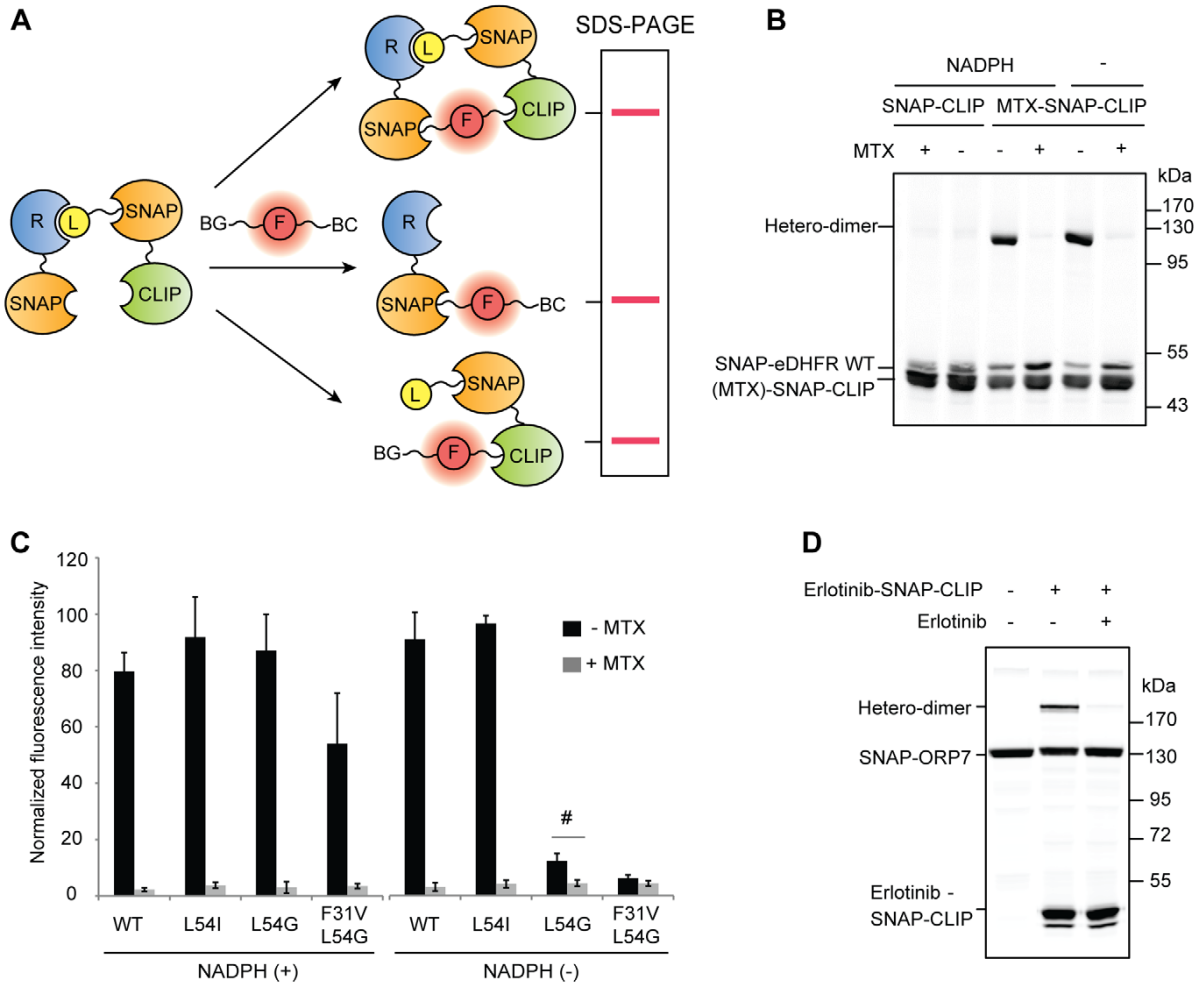
S-CROSS assay for the detection of ligand-receptor interactions. (A) Scheme of the S-CROSS assay. SNAP-receptor and ligand-SNAP-CLIP are incubated to form a complex. BG-fluorophore-BC preferentially crosslinks proteins that are in close spatial proximity. (B) SNAP-eDHFR WT (1 µM) was mixed with MTX-SNAP-CLIP or unlabeled SNAP-CLIP (1 µM) in the presence or absence of free MTX (50 µM). The experiments were performed in the absence or presence of 100 µM NADPH. Then, the mixture was treated with 2.5 µM BG-647-BC. Labeled proteins were resolved by SDS-PAGE and detected by in-gel fluorescence scanning. (C) Fluorescence signal of the hetero-crosslinking products in the presence or absence of free MTX (Mean±SD, n = 3–5). # represents P = 0.001 in paired t-test. (D) S-CROSS assay in cell extract. SNAP-ORP7 was expressed in HEK293 cells. After preparation of extract, SNAP-ORP7 was subjected to S-CROSS assay using erlotinib-SNAP-CLIP (2 µM) in the absence or presence of free erlotinib (10 µM).

Given the limited sensitivity of the SNAP-based pulldown assay, we wished to have a more sensitive binding assay than pulldown for validation purposes. S-CROSS was originally developed in our laboratory to detect protein-protein interactions [10]. In this assay, the putative interacting proteins of interest X and Y are fused respectively to SNAP-tag and CLIP-tag (another self-labeling protein which specifically reacts with benzylcytosine (BC) derivatives [22]). If the tagged proteins X and Y interact and are in close spatial proximity, they can be hetero-crosslinked using bifunctional molecules that contain both BG and BC attached to a fluorophore. The hetero-crosslinking products are then detected by SDS-PAGE followed by fluorescence scanning. To utilize this assay for the detection of ligand-receptor interactions, the ligand is attached to the SNAP moiety of a SNAP-CLIP protein, resulting in the formation of the ligand-SNAP-CLIP where CLIP tag is available for further modification with the cross-linker (Figures 1B, 6A).

Figure 6B shows a representative result of the S-CROSS assay using SNAP-eDHFR WT (1 µM) and MTX-SNAP-CLIP (1 µM). The band that migrated between 95–130 kDa appeared specifically in the lanes with MTX-SNAP-CLIP (regardless of the presence or absence of NADPH) but not in the lanes with SNAP-CLIP not attached to MTX. We therefore assigned this band to the hetero-crosslinking product between SNAP-eDHFR WT and MTX-SNAP-CLIP. Free MTX prevented the formation of the hetero-crosslinking product. The same assay was performed with all eDHFR mutants, and the quantification of the signal of the hetero-crosslinking products is presented in Figure 6C. It was possible to detect crosslinking with eDHFR L54G (without NADPH) but not with the eDHFR F31V, L54G (without NADPH). These results indicate that the upper detection limit of S-CROSS lies in the range of Kd values of at least 13 µM, which is more than 5-fold higher than that of our pulldown assays (2 µM).

Another important property of a binding assay is the possibility to perform it in cell extracts, in case that the interaction between a ligand and a receptor require additional cellular factors. To evaluate if the S-CROSS assay also is applicable in cell extracts, we used extracts of HEK293 cells transiently expressing SNAP-ORP7. Upon addition of erlotinib-SNAP-CLIP to the cell extract, a new band migrating at 189 kDa was detected (Figure 6D), indicating the formation of the hetero-crosslinking product of SNAP-ORP7 and erlotinib-SNAP-CLIP. In the presence of free erlotinib, this band was no longer detected. This experiment therefore confirmed that the S-CROSS assay is also applicable to analyze ligand-protein interactions in cell extracts.

### Cell Imaging Assay

**Figure 7:**
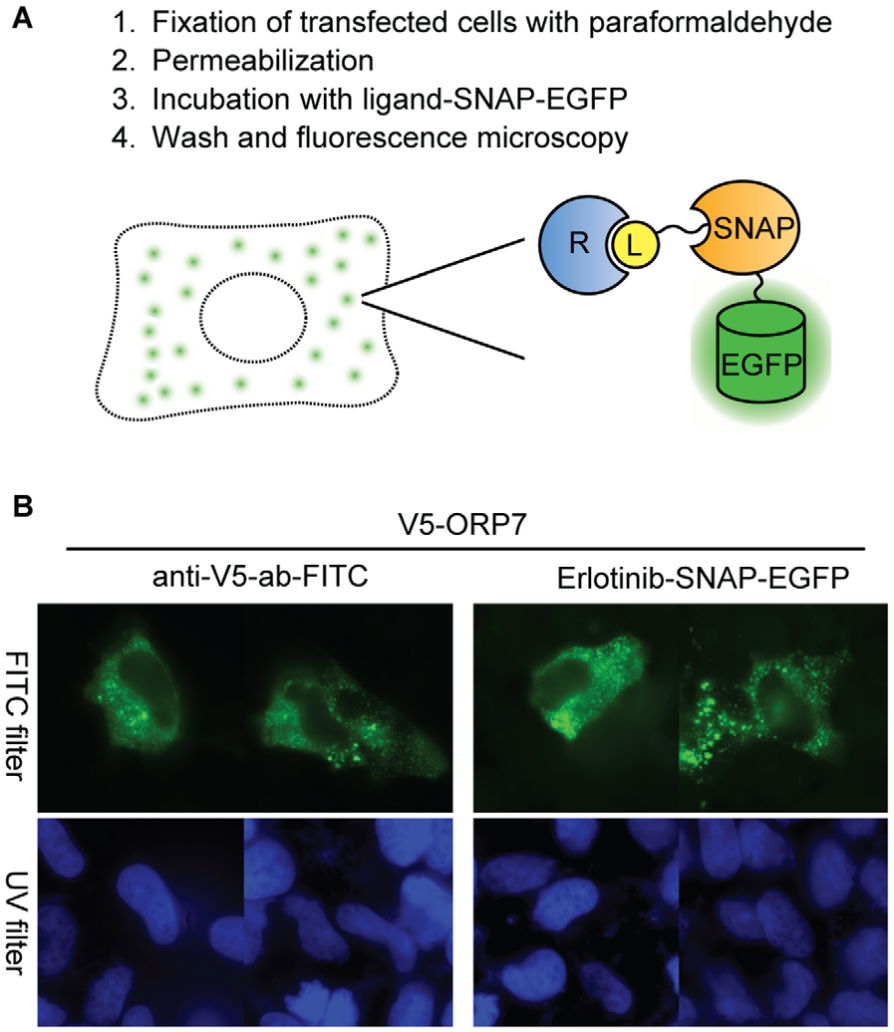
Cell imaging assay for detection of ligand-receptor interactions. (A) Scheme of the cell imaging assay. The receptor protein is transiently expressed in mammalian cells. Cells are fixed with 4% paraformaldehyde, and then permeabilized with detergent. Ligand-SNAP-EGFP is incubated with the cells. Immediately after washing, samples are analyzed with a fluorescence microscope. (B) Fluorescence micrographs of U2OS cells transiently expressing V5-ORP7 probed with FITC-conjugated anti-V5 antibody (left) and erlotinib-SNAP-EGFP (right) by cell imaging assay (FITC filter in green). Nuclear staining with Hoechst 33342 is shown in blue (UV filter).

Finally, we developed a binding assay applicable to fixed cells. Such an assay is desirable if the protein to be analyzed is insoluble or unstable in extracts. In this assay (Figure 1B, 7A), the epitope-tagged receptor protein is over-expressed in mammalian cells by transient transfection. The cells are fixed with paraformaldehyde and permeabilized with nonionic detergent. The expression of the receptor protein is then confirmed by visualization with the anti-epitope tag antibody conjugated with a fluorophore using immunofluorescence microscopy. In parallel, the ligand-protein interaction is examined with the aid of BG-derivatized ligand conjugated to EGFP via SNAP-tag. Fluorescence micrographs (anti-V5-ab-FITC) in Figure 7B show that the transiently overexpressed V5-tagged ORP7, labeled with FITC-conjugated anti-V5 antibody, is localized in the cytoplasm of the transfected cells, consistent with reported data [23]. A similar cytoplasmic localization of V5-ORP7 was visualized with erlotinib-SNAP-EGFP but not with SNAP-EGFP (data not shown). This demonstrates that it is possible to detect the erlotinib-ORP7 interaction in paraformaldehyde-fixed cells. We evaluated the generality of this method using other previously validated drug-protein interactions (PDE6D-atorvastatin, PTK2B-purvalanol B, and FYN-dasatinib). Binding of all three pairs was detected with this method (Figure S3).

## Discussion

Despite the availability of numerous conceptually different approaches to identify and characterize ligand-receptor interactions, there is a need for alternative methodologies that are both sensitive and suitable for applications with either isolated proteins or in complex mixtures. We introduce here three different assays for analyzing ligand-receptor interactions based on the specific conjugation of BG-labeled ligands to SNAP-tag fusion proteins. SNAP-tag fusions provide the ligand-protein conjugate with functionalities that either enable the detection of ligand-receptor interactions in high-throughput assays or the validation of suspected interactions in cell extracts or fixed cells. The approach is based on the assumption that the ligand can be derivatized with BG and coupled to SNAP-tag without abolishing its affinity for the receptor protein. All three assays were tested using different, previously identified ligand-protein interactions [7]; each interaction pair we tested was confirmed by our assays.

The first assay we introduce is particularly well suited for a quantitative analysis of ligand-receptor interactions. In this TR-FRET assay the ligand is coupled to a SNAP-EGFP fusion protein that functions as the FRET acceptor of a terbium-cryptate which was coupled to the receptor protein via SNAP-tag. Using this approach, we were able to detect interactions ranging from 0.4 nM to 13 µM in tracer titration experiments. It should be noted that the efficiency of the SNAP-tag labeling reaction makes a purification of the labeled SNAP-tag fusions unnecessary, thereby facilitating the implementation of the assay. Furthermore, the high sensitivity of the SNAP-based TR-FRET assay makes it well suited for high-throughput screening. The SNAP-based competition binding assay is especially useful when simple enzymatic assays are not available for the protein of interest.

The second assay we introduce is particularly well suited for a validation of suspected ligand-receptor interactions in cell lysates. It is based on a selective crosslinking (S-CROSS) assay in which the ligand is coupled to a SNAP-CLIP fusion protein and then selectively crosslinked to the suspected target protein that is expressed as a SNAP-tag fusion protein. The key features of S-CROSS are its high sensitivity, permitting the detection of Kd values above 10 µM in favorable cases, as well as the fact that it can be performed in cell lysates. This makes it an attractive alternative to more traditional pulldown assays, even if the latter can also be utilized to detect endogenous proteins.

The third assay is a cell imaging assay utilizing fixed cells and a ligand bound to a SNAP-EGFP fusion protein for fluorescence staining. The assay appears particularly well suited for proteins that cannot be (easily) expressed as soluble and stable proteins. The cell imaging assay can be performed with a small number of cells and is experimentally simple and fast.

In addition to these three assays, we previously introduced a SNAP-based yeast three-hybrid system as well as a SNAP-based pulldown assay. The synthesis of a BG derivative of a given ligand therefore creates access to a powerful toolbox that permits: (i) the search for the target proteins of ligands, (ii) the validation of identified interactions in cell lysates or fixed cells, (iii) the quantification of ligand-receptor interactions, and (iv) the establishment of robust assays for high-throughput application. Furthermore, the possibility to express numerous other SNAP-tag fusion proteins should allow the design of additional assays. Ligand-protein conjugates should therefore become important tools in the analysis of ligand-receptor interactions.

## Materials and Methods

### Reagents

The synthesis of methotrexate-BG (MTX-BG) and BG-erlotinib using O6-benzylguanine-(PEG)_4_-NH_2_ was described previously [24]. SNAP-Cell Fluorescein (BG-fluorescein), SNAP-Cell TMR-Star (BG-tetramethylrhodamine), SNAP-Surface 647 (BG-647) were provided by NEB. BG derivatized with terbium cryptate (BG-Tb, SNAP-Lumi4-Tb) was provided by Cisbio Bioassays. Stocks of all compounds were prepared in DMSO and stored at −20°C.

### Cell Culture

U2OS cells (ATCC, HTB-96) were cultured in DMEM supplemented with 10% fetal bovine serum [7]. Suspension-adapted HEK-293E cells obtained from David Hacker (EPFL, Switzerland) were cultured in EX-CELL293 serum-free medium (SAFC Biosciences) with 6 mM L-glutamine [25].

### Plasmids

The *E.coli* expression plasmid pET-His-SNAP-eDHFR was constructed by DNA recombination with the destination plasmid pET-His-SNAP/DEST and gateway entry plasmid pENTR221-eDHFR using LR clonase II Enzyme mix (Invitrogen). pET-His-SNAP/DEST was constructed by insertion of the DNA sequence of SNAP-tag between the DNA sequence of the hexa-histidine tag and the DNA sequence of attR1 site of pDEST17 (Invitrogen). pENTR221-eDHFR (WT and mutants) was constructed by DNA recombination between pDONR221 (Invitrogen) and PCR products containing ORF of eDHFR flanked with attB sites (pGAD-HA-eDHFR was used as PCR template [7]) using BP clonase II (Invitrogen). The *E.coli* expression plasmid pET51b-SNAP-EGFP (A207K) expresses SNAP-EGFP (A207K) with hexa-histidine and strep-tags at amino and carboxyl terminus, respectively. EGFP (A207K) mutant (corresponds to YFP (A206K) in the reference [26]) was chosen to prevent possible homo-dimerization of EGFP at high protein concentration. The EGFP sequence originally derived from pEGFP-TUB (CLONTECH) was replaced with the CLIP sequence of pET51b-SNAP-CLIP [27]. Then, the A207K mutation was introduced with overlap PCR. pET51b-SNAP-EGFP was used for the cell imaging assay, while pET51b-SNAP-EGFP (A207K) was used for the TR-FRET assay. Construction of pcDNA3.1-V5-SNAP/DEST and pcDNA3.1-V5-ORP7 was previously described [7]. Construction of pcDNA3.1-Strep-SNAP/DEST was performed as described in construction of pcDNA3.1-Flag-SNAP/DEST but with oligonucleotides containing single strep-tag sequence (WSHPQFEK) [7].

### Preparation of Recombinant Proteins

SNAP-eDHFR (WT and mutants) were expressed from pET-His-SNAP-eDHFR in *E.coli* BL21, and purified by Ni-NTA affinity chromatography. These proteins were further purified with a Superdex S200 10/300GL column (GE healthcare) in buffer A (50 mM Hepes-NaOH pH 7.4, 150 mM NaCl, 10% glycerol, and 1 mM DTT), and stored at −80°C. SNAP-ORP7 tagged with strep-tag at the amino terminus was expressed as described by Backliwal et al [25], [28]. Two days after transfection, HEK293 cells were collected and lysed in the presence of 10 cell pellet volume of lysis buffer (50 mM Tris-HCl pH 7.9, 150 mM NaCl, 5 mM EDTA, 1% Triton X-100, and protease inhibitor cocktail (Complete Mini, Roche)) at room temperature for 15 min. The extract was cleared by centrifugation at 15 krpm for 15 min at 4°C, and the supernatant was dialyzed against buffer (0.1 M Tris-HCl pH 7.9, 150 mM NaCl, and 1 mM EDTA) using 14 kDa MWCO dialysis membrane (SPECTRUM). The extract was subjected to affinity chromatography using Strep-tactin sepharose column (IBA) according to the manufacturer’s instructions. Purified SNAP-ORP7 was dialyzed against buffer A. Purity of SNAP-ORP7 prepared in this way was >90%, as determined by SDS-PAGE and Coomassie brilliant blue (CBB) staining. SNAP-EGFP (A207K) was expressed from pET51b-SNAP-EGFP (A207K) and induced in *E.coli* BL21 using auto-induction medium ZYP-5052 [29], purified with a Ni-NTA affinity column and subsequent Strep-tactin sepharose column chromatography, and then dialyzed against buffer A. The purity, estimated by SDS-PAGE and CBB staining, was more than 95%. SNAP-CLIP was expressed from pET51b-SNAP-CLIP [27] and purified as SNAP-EGFP.

### Determination of Concentration of Reagents

The concentration of MTX and MTX-BG was determined based on ε (373 nm) = 7800 cm^−1^ M^−1^. The protein concentrations were determined using the absorbance at 280 nm based on the extinction coefficient (ε = 56170 cm^−1^ M^−1^ of SNAP-eDHFR, ε = 46300 cm^−1^ M^−1^ of SNAP-CLIP) calculated from the amino acid sequences. The concentration of SNAP-EGFP (A207K) was determined based on ε (488 nm) = 68000 cm^−1^ M^−1^ of EGFP (A207K). This extinction coefficient was determined experimentally by comparison with SNAP-EGFP WT (ε (488 nm) = 55000 cm^−1^ M^−1^) using the absorbance at 280 nm and 488 nm of the two proteins. The molar ratio of active SNAP and fluorescent EGFP of the purified SNAP-EGFP (A207K) was measured as follows. SNAP-EGFP was labelled with 2-fold excess BG-647 for complete labeling, then unreacted substrate was removed by NAP5 gel filtration column (GE healthcare). The absorbance of the labelled protein at 488 nm and 650 nm was measured, and concentration of EGFP (A207K) (ε(488 nm) = 68000 cm^−1^ M^−1^) and fluorophore 647 (ε 650 nm) = 250000 cm^−1^ M^−1^) was calculated. Measured ratio was approximately 1. The concentration of SNAP-ORP7 was determined by labeling with BG-647 and subsequent in-gel fluorescence scanning (BIO-RAD) and by comparison to reference protein samples using the Quantity One software (BIO-RAD).

### TR-FRET Assay

SNAP labeling experiments with BG-substrate were performed at room temperature for 1 h. Partial labeling of the SNAP-receptor with BG-Tb (Cisbio Bioassays) was performed by mixing 1 µM receptor protein and 0.5 µM BG-Tb in buffer A supplemented with 0.5 mg/ml BSA and 0.05% Triton X-100. We performed partial labeling with BG-Tb to avoid additional purification steps to remove excess BG-Tb. SNAP-EGFP (A207K) was completely labeled with 2-fold molar excess of BG-ligand in buffer A. Free BG-ligand was removed by NAP5 gel filtration column. The concentration of ligand-SNAP-EGFP (A207K) was determined using absorbance at 488 nm (ε (488 nm) = 68000 cm^−1^ M^−1^). The aliquots of receptor and tracer were stored at −80°C. The yield of SNAP labeling was characterized as follows. The purified ligand-labeled SNAP fusion protein was mixed with 10 µM BG-fluorescein or BG-tetramethylrhodamine (TMR), both of which have fast reaction rates. Under these conditions, all the unreacted SNAP is then fluorescently labeled with the fast-reacting BG-fluorophore. The fluorescently labeled SNAP fusion protein and protein standards of SNAP fusion protein labeled with same BG-fluorophore for quantification were resolved by SDS-PAGE and detected by in-gel fluorescence scanning.

In the titration assay, SNAP-eDHFR partially labeled with BG-Tb was mixed with 3-fold dilution series of MTX-SNAP-EGFP or SNAP-EGFP blocked with BG in buffer A supplemented with 0.5 mg/ml BSA and 0.05% Triton X-100 in the absence or presence of 100 µM NADPH in a final volume of 70 µl. A receptor concentration was lower than the corresponding Kd value to prevent ligand depletion. Aliquots of 20 µl were transferred in 3 wells of a black 384 well plate (Corning 3820). Signal was measured 1 h later in the titration assay by EnVision (PerkinElmer). An excitation filter of 320 nm and emission filters of 486 nm and 510 nm were used. Delay time and time windows were set to 120 µs and 400 µs, respectively.

In the competition assay, 10 µl of pre-formed mixture of BG-Tb labeled SNAP-eDHFR and MTX-SNAP-EGFP in 2×buffer E (0.1 M Hepes-NaOH pH 7.4, 0.3 M NaCl, 1 mg/ml BSA, 0.1% TritonX-100, 2 mM DTT and 0.1 mM NADPH) were transferred in a well of 384 well plates, and then 10 µl of 5-fold serial dilutions of compounds prepared in water were added in quadruplicate. Plates were sealed and left at room temperature in the dark for 4 days (SNAP-eDHFR WT) and 3 h (SNAP-eDHFR L54I and L54G) until measurement of signal. Time course experiments showed that it took 4 days for SNAP-eDHFR WT and 2 h for SNAP-eDHFR L54I and L54G to reach equilibrium (Figure S4). Z-factor was calculated using water and 1 µM trimethoprim as negative and positive control, respectively, using the equation previously described [20]. DHFR enzymatic assay was performed by following decrease of absorbance at 340 nm for 1 h at room temperature in transparent 96 well plates. 50 µl of enzyme mixture containing 0.4 nM SNAP-eDHFR WT in 4 × buffer E was added to a well. Then, 50 µl of 5-fold serial dilution of compounds prepared in water was added. The reaction was started by addition of 100 µl of 80 µM dihydrofolic acid in 10 mM Hepes-NaOH (pH 7.4). The assays were performed in triplicate. Data points for 1 h were used for determination of enzyme activity.

### Curve Fitting

The Kd value of the tracer for SNAP-eDHFR was calculated by fitting to the full equation of single site binding with the effect of nonspecific binding [18].
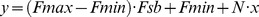
with



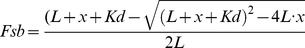



In the equations, the dependent variable x is the concentration of the tracer and the dependent variable y is the emission signal at 510 nm. L is the total concentration of the receptor and Kd is the dissociation constant. N is a parameter of nonspecific interaction between the tracer and the receptor and/or between the fluorophore and Tb. Fmax and Fmin are respectively the maximum and minimum values of emission signal when N = 0. Global fitting was performed for Figure 2B using OriginPro 8.5 software. Kd value of SNAP-EGFP (A207K) blocked with BG for SNAP-eDHFR was fixed to 100 µM and not infinite, since the statistical evaluation was more satisfactory. The Kd values with asterisk in Figure 2 and Table 1 were too high to precisely fit without restriction of a parameter Fmax, which was taken from higher affinity mutants with the assumption that Fmax is the same for SNAP-eDHFR WT and mutants.

The Kd value of competitor for SNAP-eDHFR was calculated by fitting to full equation of single site competitive binding [19]. In the competition assay, emission ratio (defined as emission signal at 510 nm from EGFP normalized with emission signal at 486 nm from Tb-cryptate) was used to minimize the inner filter effect caused by higher concentration of some compounds.
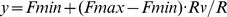
with



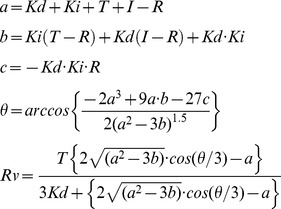



In the equations, the dependent variable I is the concentration of the competitor and the dependent variable y is the emission ratio. Kd is the dissociation constant of the tracer for the receptor, Ki is the dissociation constant of the competitor for SNAP-eDHFR, T is the total tracer concentration, and R is the total receptor concentration. Fmax and Fmin are the maximum and minimum values of emission ratio, respectively.

### Pulldown Assay

SNAP-based pulldown assay was performed as previously described [7], but with modification of the detection method for the target proteins. SNAP labeling was performed for 20–30 min at room temperature. 20 µl of 20% sepharose 4B slurry in buffer B (50 mM Tris-HCl pH 7.9 and 0.5 M NaCl) were mixed with 60 µl of *E. coli* extract containing bead-saturating amounts of GST-SNAP and incubated for 30 min at 4°C with rotation. The beads were then washed twice with 1 ml buffer A and resuspended in 50 µl of the same buffer. The immobilized GST-SNAP was labeled with MTX-BG (10 µM) with rotation. After the incubation, unreacted GST-SNAP was blocked with an excess of O^6^-BG (SIGMA). After blocking, the beads were washed twice with 200 µl buffer A. The recombinant SNAP-eDHFR (1 µM) was labeled with 10 µM BG-647 in buffer A to allow in-gel visualization of the proteins. Then, the unreacted SNAP was blocked by addition of 100-fold excess O^6^-BG. 5 µl of each labeled protein were used for the input (10% of the total). Immobilized MTX-SNAP-GST and SNAP-GST were incubated with 647-SNAP-eDHFR in the presence and absence of 100 µM NADPH in a final volume of 50 µl. The beads were incubated for 2 h at 4°C with rotation. After washing twice with 200 µl buffer A, the bound proteins were eluted with 20 µl of 50 mM Tris-HCl pH 7.9 supplemented with 10 mM reduced glutathione for 15 min at room temperature. The eluted proteins were resolved in 12% SDS polyacrylamide gels and visualized by in-gel fluorescence scanning. The fluorescence intensity of the bands was quantified with the Quantity one software.

### S-CROSS Assay

SNAP-CLIP (5 µM) in buffer A was labeled with MTX-BG (10 µM) for 1 h at room temperature. Then free MTX-BG was removed by NAP-5 column purification using buffer A. MTX-SNAP-CLIP (1 µM) and SNAP-eDHFR (1 µM) were mixed in the absence and presence of 100 µM NADPH and/or 50 µM free MTX in buffer A in a final volume of 20 µl for 1 h at room temperature. Then the cross-linker BG-Cy5-BC [10] (2.5 µM) was added and the reactions were incubated for 1 h at 37°C. The reactions were stopped by addition of SDS sample loading buffer and incubation for 5 min at 95°C. The proteins were analyzed as described for pulldown assay.

### Cell Imaging Assay

U2OS cells in a µ-Dish (Ibidi) were transfected with pcDNA3.1-V5-ORP7 using Lipofectamine 2000 reagent (Invitrogen) and cultured for 48 h. Following procedures were performed at room temperature. The cells were washed twice with PBS and fixed with 4% paraformaldehyde pH 7.0 in PBS for 10 minutes. After washing three times with PBS, the cells were permeabilized with 0.1% Triton X-100 in PBS for 5 minutes. The cells were then washed twice and blocked with 1% non-fat milk in PBS for 30 minutes. Expression of the V5-tagged protein was confirmed by immunofluorescence microscopy with the α-V5-FITC conjugated antibody (Invitrogen), diluted 1∶1000 in PBS, for 2 h in the dark. SNAP-EGFP was completely labeled with BG-erlotinib as described above and eluted in PBS. The fixed cells were incubated for 2 h with 250 µL of 500 nM erlotinib-SNAP-EGFP or unlabeled SNAP-EGFP in 1% non-fat milk-PBS. Hoechst 33342 (0.1 µg/ml) was added 10 minutes before the imaging for nuclear staining. The cells were then washed with PBS 3 times and imaged within 30 min after washing using a 120× objective with a Zeiss Axiovert 200 inverted microscope equipped with an AxioCam MR digital camera (Zeiss).

## Supporting Information

Figure S1 **SNAP-based TR-FRET competition assay using MTX and MTX-BG as competitors in the presence of 100 µM NADPH.** Kd values and the standard error of the mean are shown in the graph.
(TIF)

Figure S2 **Z-factor of the assay setup of** **Figure 3B** **.** Emission ratio was measured in the absence and presence of 1 µM trimethoprim in the assay setup of Figure 3C (concentrations of receptor and tracer are indicated), and plotted against number of wells. Indicated Z-factors were calculated from the data.
(TIF)

Figure S3 **Cell imaging assay using validated drug-receptor pairs.** (A) U2OS cells were transfected with a plasmid which express V5 tagged PDE6D. One day after transfection, cells were fixed with 4% paraformaldehyde, and permeabilized with 0.1% Tritin X-100. After washing, the cells were incubated with anti-V5-antibody conjugated with FITC or 100 nM atorvastatin-SNAP-EGFP. Fluorescence images (GFP filter) were taken within 30 min after washing of the cells. (B) (C) Cell imaging assay using pairs of purvalanol B-PTK2B and dasatinib-FYN, respectively.
(TIF)

Figure S4 **Time-course experiments of SNAP-based TR-FRET competition binding assay.** (A) A mixture containing 90 pM SNAP-eDHFR WT and 1.2 nM MTX-SNAP-EGFP were prepared as described in materials and methods. At indicated time after addition of methotrexate, emission signal at 510 nm and 486 nm wavelengths was measured. Emission ratio (emission signal at 510 nm divided by emission signal at 486 nm) was plotted against concentration of methotrexate. (B) The same experiment as (A) was performed in parallel with 90 pM of SNAP-eDHFR L54I and 2.2 nM of MTX-SNAP-EGFP.
(TIF)
