# Supplementary figures and images for: Exploiting Ligand-Protein Conjugates to Monitor Ligand-Receptor Interactions
Source: PLoS One. 2012 May 31;7(5):e37598. doi: 10.1371/journal.pone.0037598 (PMC3365113; doi:10.1371/journal.pone.0037598)

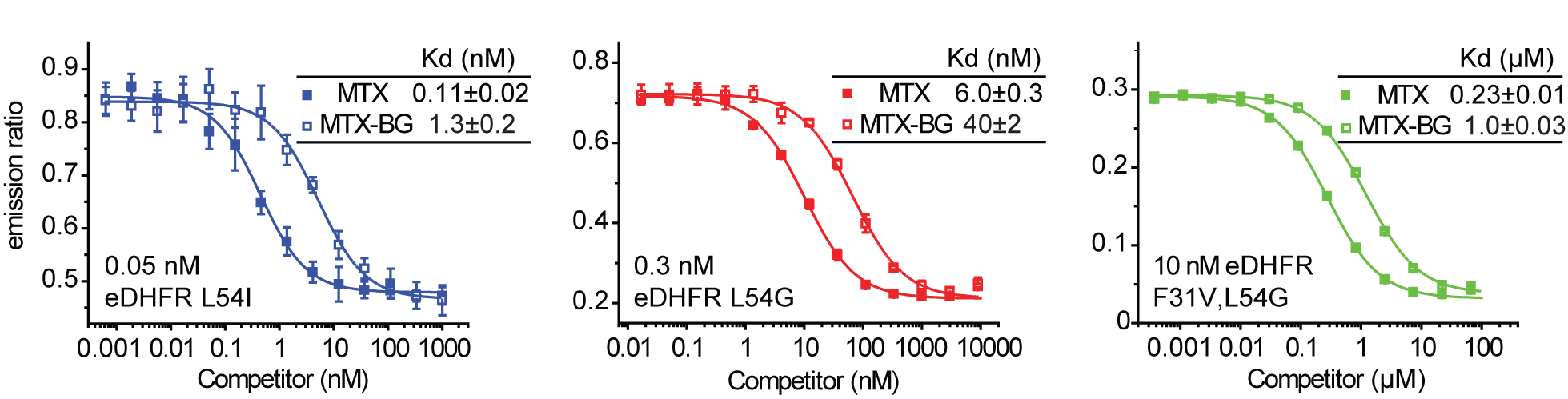

Supplement: Figure S1 — SNAP-based TR-FRET competition assay using MTX and MTX-BG as competitors in the presence of 100 µM NADPH. Kd values and the standard error of the mean are shown in the graph. (TIF) [file pone.0037598.s001.tif]

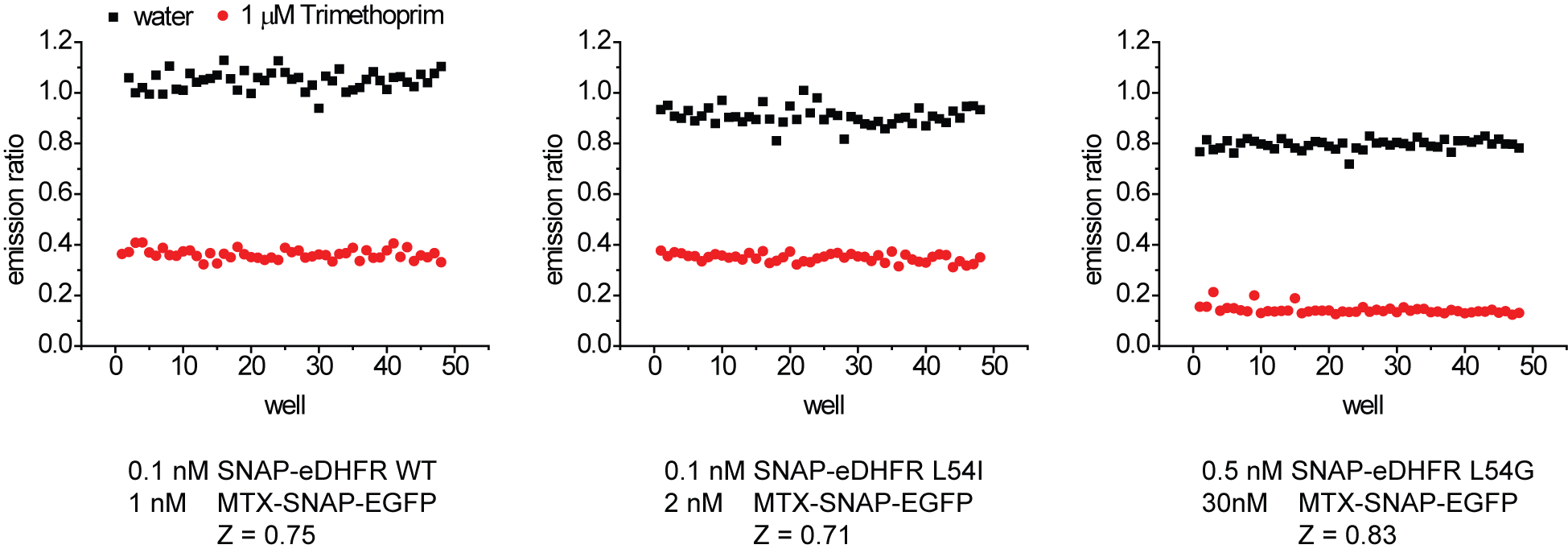

Supplement: Figure S2 — Z-factor of the assay setup of Figure 3B . Emission ratio was measured in the absence and presence of 1 µM trimethoprim in the assay setup of Figure 3C (concentrations of receptor and tracer are indicated), and plotted against number of wells. Indicated Z-factors were calculated from the data. (TIF) [file pone.0037598.s002.tif]

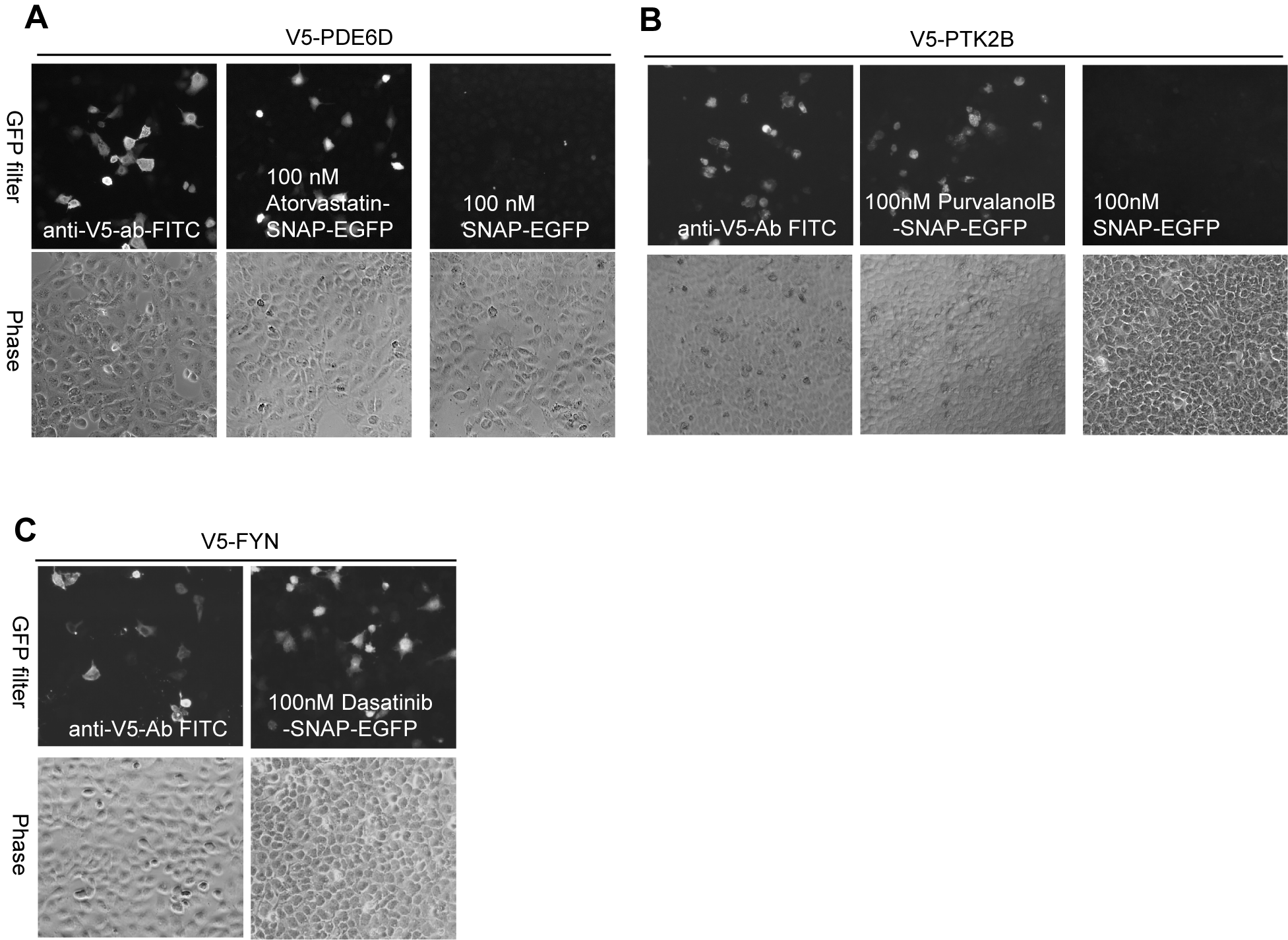

Supplement: Figure S3 — Cell imaging assay using validated drug-receptor pairs. (A) U2OS cells were transfected with a plasmid which express V5 tagged PDE6D. One day after transfection, cells were fixed with 4% paraformaldehyde, and permeabilized with 0.1% Tritin X-100. After washing, the cells were incubated with anti-V5-antibody conjugated with FITC or 100 nM atorvastatin-SNAP-EGFP. Fluorescence images (GFP filter) were taken within 30 min after washing of the cells. (B) (C) Cell imaging assay using pairs of purvalanol B-PTK2B and dasatinib-FYN, respectively. (TIF) [file pone.0037598.s003.tif]

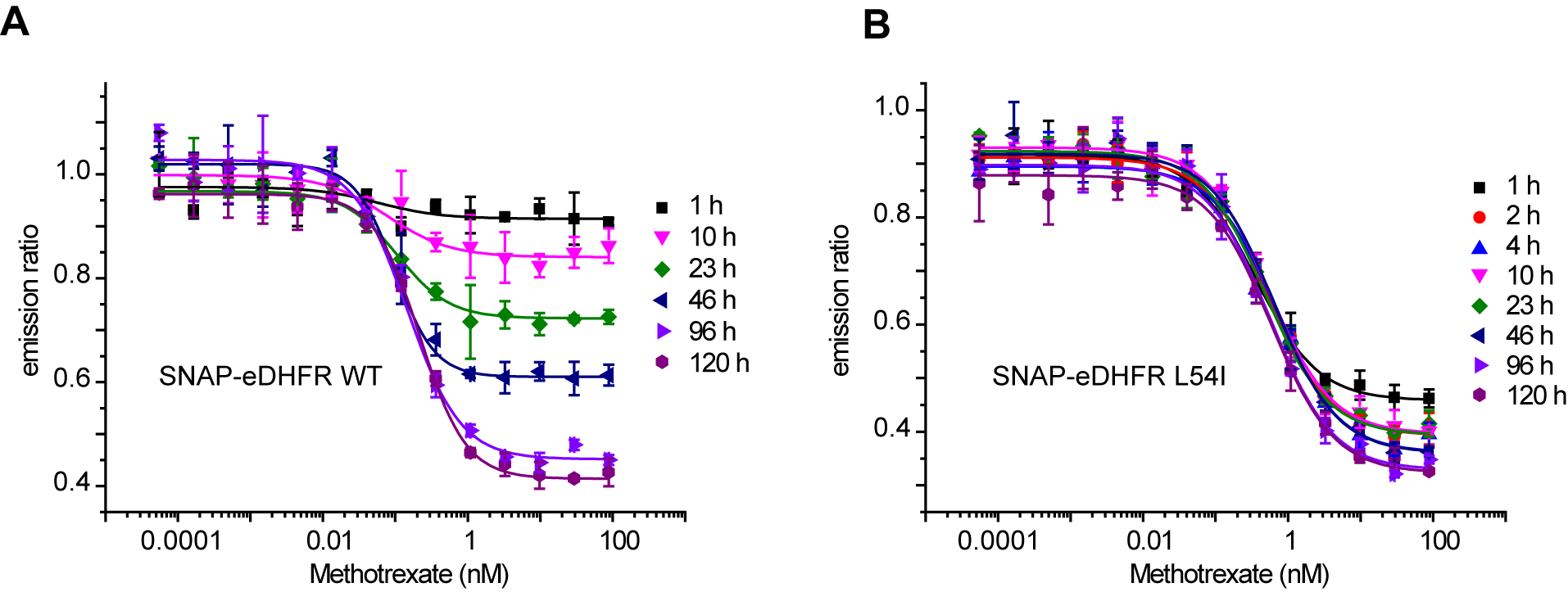

Supplement: Figure S4 — Time-course experiments of SNAP-based TR-FRET competition binding assay. (A) A mixture containing 90 pM SNAP-eDHFR WT and 1.2 nM MTX-SNAP-EGFP were prepared as described in materials and methods. At indicated time after addition of methotrexate, emission signal at 510 nm and 486 nm wavelengths was measured. Emission ratio (emission signal at 510 nm divided by emission signal at 486 nm) was plotted against concentration of methotrexate. (B) The same experiment as (A) was performed in parallel with 90 pM of SNAP-eDHFR L54I and 2.2 nM of MTX-SNAP-EGFP. (TIF) [file pone.0037598.s004.tif]
